# Host, Weather and Virological Factors Drive Norovirus Epidemiology: Time-Series Analysis of Laboratory Surveillance Data in England and Wales

**DOI:** 10.1371/journal.pone.0006671

**Published:** 2009-08-24

**Authors:** Ben Lopman, Ben Armstrong, Christina Atchison, Jim J. Gray

**Affiliations:** 1 Gastrointestinal, Emerging and Zoonotic Infections Department, Centre for Infections, Health Protection Agency, London, United Kingdom; 2 Department of Epidemiology and Population Health, London School of Hygiene and Tropical Medicine, London, United Kingdom; 3 Department of Public Health and Policy, London School of Hygiene and Tropical Medicine, London, United Kingdom; 4 Virus Reference Department, Centre for Infections, Health Protection Agency, London, United Kingdom; University of Oxford, United Kingdom

## Abstract

Norovirus, the most commonly identified cause of both sporadic cases and outbreaks of infectious diarrhoea in developed countries, exhibits a complex epidemiology and has a strong wintertime seasonality. Viral populations are dynamic and evolve under positive selection pressure.

**Methods:**

Time series-adapted Poisson regression models were fitted to daily counts of laboratory reports of norovirus in England and Wales from 1993 to 2006.

**Findings:**

Inverse linear associations with daily temperature over the previous seven weeks (rate ratio (RR) = 0.85; 95% CI: 0.83 to 0.86 for every 1°C increase) and relative humidity over the previous five weeks (RR = 0.980; 95% CI: 0.973 to 0.987 for every 1% increase) were found, with temperature having a greater overall effect. The emergence of new norovirus variants (RR = 1.16; 95% CI: 1.10 to 1.22) and low population immunity were also associated with heightened norovirus activity. Temperature and humidity, which may be localised, had highly consistent effects in each region of England and Wales.

**Conclusions:**

These results point to a complex interplay between host, viral and climatic factors driving norovirus epidemic patterns. Increases in norovirus are associated with cold, dry temperature, low population immunity and the emergence of novel genogroup 2 type 4 antigenic variants.

## Introduction

Noroviruses are the most commonly identified cause of acute gastroenteritis amongst both sporadic community cases and outbreaks.[1], [2] Noroviruses are single stranded RNA viruses and members of the *Caliciviridae* family. There are two main genogroup of viruses causing disease in humans, with substantial genetic diversity between and within genogroups. Although norovirus gastroenteritis tends to be short-lived and resolves without medical intervention in otherwise healthy individuals, evidence suggest that infections may be more severe in vulnerable populations.[3] In industrialised countries, outbreaks frequently occur in healthcare settings,[4] where economic impact may be substantial and associated deaths may occur amongst the elderly.[5] In developing countries, malnourished children or those without access to effective healthcare may suffer substantial morbidity and mortality to norovirus,[6] though this burden is yet to be accurately quantified.

Noroviruses, like many other respiratory and gastrointestinal viruses, exhibit wintertime seasonality in temperate climates.[7] However, norovirus epidemic patterns are highly irregular. Unlike rotavirus, the norovirus peak frequently shifts by calendar weeks or months between seasons. And, unlike influenza A virus, a substantial genetic diversity in viral populations circulate concomitantly. Because norovirus cannot be readily cultured *in vitro* and there are no animal models of infection, studies of virus survival and transmission under different environmental conditions cannot be performed.[8] Low relative humidity and temperature (i.e. cool and dry conditions) have been identified to promote transmission of respiratory viruses in the laboratory as well as in human populations.[9], [10] Similar studies of enteric virus transmission have shown mixed results with some suggesting low and others suggesting higher temperatures associated with transmission.

Given the high variability of norovirus seasonality, it is unlikely that seasonal environmental factors alone govern transmission patterns of disease. Immunity to norovirus infection and disease is short lived (somewhere between 2 and 6 months) and heterotypic protection is limited.[11] Norovirus is highly infectious. Due to these combined factors, nearly all children will have had at least one norovirus infection by their fifth birthday but infections and disease occur throughout life as immunity wanes and new antigenic types are encountered. Indeed, noroviruses are constantly evolving, with the most common group of viruses (genogroup II genotype 4) under positive selective pressure – whereby immune escape variants (an adaptive trait) are selected for.[12] New variants with antigenic changes may escape population immunity. The emergence of such variants has been shown to be associated with substantial increases in cases worldwide.[13]

Using meteorological, viral evolution and norovirus activity data from across England and Wales, this study aims to address how weather, levels of population immunity, and the emergence of new genogroup 2 genotype 4 noroviruses affect norovirus epidemic patterns.

## Materials and Methods

### Data

The Health Protection Agency collects data from laboratories around England and Wales on reports of pathogens identified in faecal samples from infected patients with gastrointestinal symptoms.[14] Specimens are taken for testing by investigation public health or infection control teams in outbreak situations or, less commonly, by physicians from patients consulting for gastroenteritis. Only a small fraction – estimated at 1/300 to 1/1500 - community cases are reported to national surveillance.[15] Daily numbers of norovirus reports from 1993 to 2006 were extracted from the national database. From this period, there were a total of 35210 norovirus laboratory reports with known specimen date. Laboratory report data are collected from sporadic cases presenting to physicians and outbreaks investigated by public health bodies or hospital infection control teams. It is not known whether individual specimens are outbreak associated or not. Data including source laboratory, age of case and detection method are requested but often incomplete. For all analyses, the date nearest to the patient's date of onset was used, which was usually the date the specimen was received at the laboratory.

All meteorological data were obtained from the UK Met Office. Central England Temperature (CET) is an aggregate variable that represents temperature in the Midlands region of England, which is highly correlated with temperature in other regions of England.[16] Similarly, a temporal indicator of relative humidity was constructed from population-weighted measurements from individual weather stations across England and Wales. Preliminary analysis showed no evidence for association of precipitation with norovirus laboratory reports and precipitation was excluded from further analysis.

### Statistical methods

The central questions of this study are (1) how do temperature and relative humidity on day *x* affect norovirus reports on day(s) *x+t_0..n_* (2) how does the size of last year's epidemic and (3) the emergence of new genogroup 2 genotype 4 noroviruses affect the size of this year's epidemic?

Regression techniques adapted for analysis of time-series data (by incorporating lag effects, accounting for background seasonality, auto-correlation, overdispersion) were used to model the relationship between temperature, relative humidity, population immunity and emergence of new virus variants on norovirus reports after adjusting from other seasonal and temporal confounding factors. By controlling for background seasonality and other nuisance variables, these models estimate the short term effects of variables of interest (weather, immunity and virus evolution). Long-term trends and background seasonal patterns are accounted for as part of the confounder model so that regular patterns (i.e. cold weather and high norovirus incidence both occur in winter) are not inferred to be causal. All models were fitted using STATA 10.0. (STATA Corp LP, College Station Texas). Poisson regression models were fitted; parameters and standard errors were estimated using standard maximum likelihood estimation techniques. To account for overdispersion (deviance = 2.39 in the final model) in the norovirus report data, standard errors were scaled using square root of Pearson chi-squared goodness of fit statistic.[17] The model was built in a stepwise fashion by first constructing the confounder model, then adding the variables of interest (lagged weather variables, population immunity, new virus variants). Autocorrelation was accounted for in the final model (Figure S1). We then investigated if there were more complicated (non-linear) associations between weather and norovirus and performed a sensitivity analysis.

#### Confounders

The number of norovirus reports increased over the study period, which may be due to reasons other than a true increase in the number of infections. To account for the general secular trend, a time polynomial of increasing order was added sequentially to the model. Norovirus diagnostics improved over the study period which likely resulted in increased reporting.[18] To account for this, a term representing the proportion of diagnoses each year made by molecular techniques (PCR or ELISA, as opposed to electron microscopy) was included, which increased from zero in 1993 to 99% in 2006. Both the time polynomial and the diagnostic indicator variable significantly improved the model suggesting that both secular trends and diagnostic improvements affected the surveillance data over the study period. However, it is not possible to separate the influence of each, nor are we interested *per se* in their exact effect. Rather, they are treated as ‘nuisance’ variables in the confounder model. Weekends and public holidays were adjusted for by including dummy indicator terms in order to control for the artefactual drop in reporting that occurs on these days.

The explanatory variables of interest (temperature and humidity) are seasonal and have a similar periodicity to norovirus incidence, and we wanted our estimates of association to be robust to the possible presence of other unmeasured seasonal factors (for example, behaviour such as people spending more time indoors during winter or aspects of weather such as levels of UV). The effects of such unmeasured seasonal factors was therefore accounted for by including Fourier terms – which are linear combinations of sine and cosine functions of date. The number of pairs of Fourier terms (annual cycles and harmonics) defines the complexity of the annual pattern modelled. We used seven– the number that which minimised the Akaike's Information Criteria (AIC).

#### Weather

There may be a delay between a climatic event (e.g. drop in temperature) and an effect on laboratory reports for two reasons. First, there are delays between when an infection occurs in the community and when a specimen is received at the laboratory. Second, if a climatic event results additional cases within one generation of infection, those additional cases may be sources of further chains of transmission; so the direct effect from an isolated event may have an impact for many generations of infection. To account for this delayed effect, variables of lagged temperature, relative humidity and rainfall were included in the model. Variously lagged variables were introduced to regressions sequentially, all models controlling for trend, improving diagnostics, background seasonality, bank holidays and weekends. Specifically lags were added one day at a time, to create a variable representing a cumulative uniformly distributed lag.[19] For example, the cumulative lagged variable at day 10 was the mean of temperature on that day and the nine previous day's temperature. Temperature and relative humidity lags appeared to be linear, i.e. each additional day of lag resulted in a linear increase in the rate ratio. The optimum lag was selected when the rate ratio levelled off, seven weeks for temperature and five weeks for relative humidity.

#### Population Immunity

**Figure 1:**
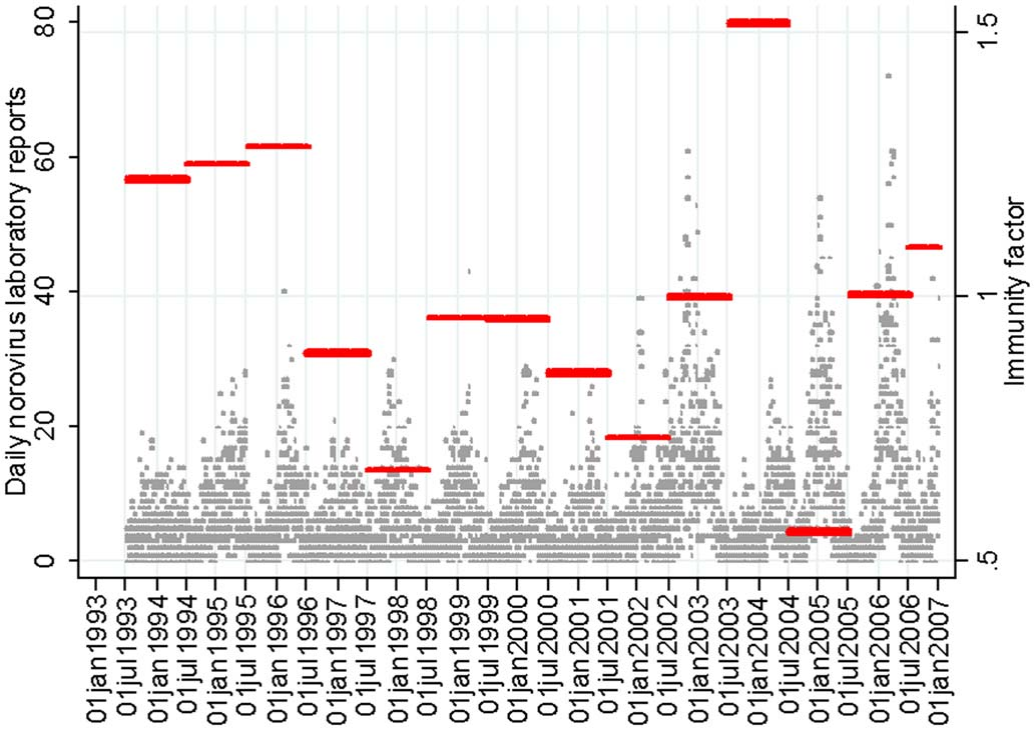
Annual calculated norovirus immunity factor. Grey dots are number of daily laboratory reports to the Health Protection Agency from 1993 to 2007. Red bars are the calculated ‘immunity factor’ which represents the level of population immunity calculated based on the size of last year's season. An immunity factor of 1 indicates a typical season in the previous year; greater than 1 indicated that last year was a larger than normal season.

To address question (2) above, a variable was constructed to represent the level of population immunity (Figure 1). This was based on the size of the previous year's epidemic. This is based on the assumption that if there were many infections in year *x*, there would be relatively more population immunity in year *x+1*. This would be true if immunity lasted 1 year and there was one antigenic type of norovirus. Both of these assumptions are a simplification. Homotypic immunity lasts approximately 6 months and there are multiple circulating antigenic types. In effect, we assume that at least a proportion of individuals infected in year *x* are immune in year *x+1*. Secondly – although norovirus are antigenically diverse – genogroup 2 genotype 4 viruses predominate. Therefore, our population immunity factor describes immunity with respect to the predominant antigenic type. [In fact, genogroup 2 genotype 4 viruses have evolving antigenicity, which we model using a separate variable as described in the paragraph below.] Because there is a secular increase in norovirus laboratory reporting. the size of last year's epidemic was normalised against the size of the three previous years as follows:
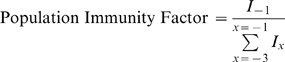
where *I* is the number of cases in the previous year *x*. A factor of 1 indicated that last year's epidemic was of expected size, with vales greater than 1 indicating a larger-than-normal epidemic in the previous year. Population immunity was modelled as a continuous variable.

#### Viral evolution

**Figure 2:**
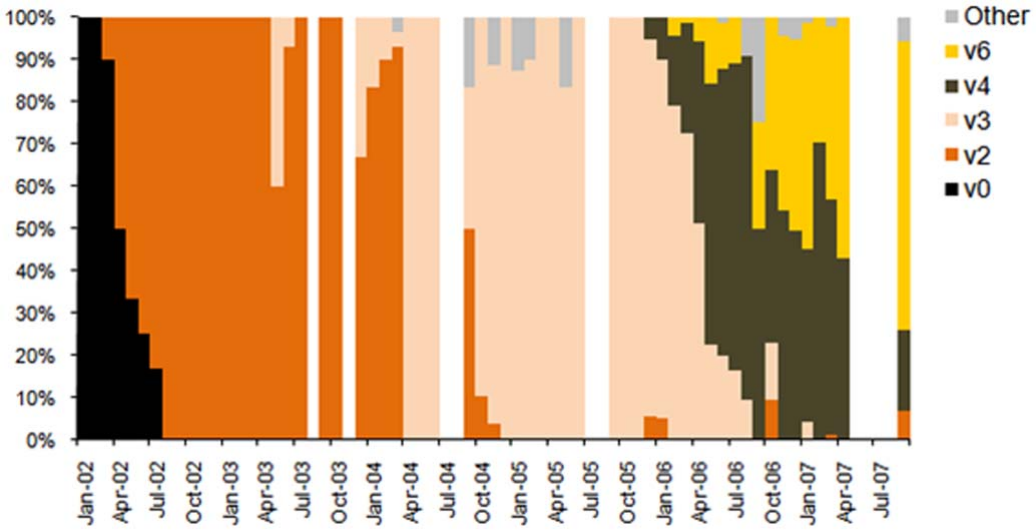
Changes in the genetic populations of norovirus genogroup 2 genotype 4 variants based on sequencing of the capsid region, 2002–2007. Based on sequencing results from the Health Protection Agency Enteric Virus Unit structured strain surveillance (n = 1378 viruses sequenced from 2002 to 2007). Strains were assigned to a variant group according to conserved nucleotides at positions 18 (A or G), 26 (G, A or C) and 43 (A or G) of the gene encoding the capsid. Variants were numbered chronologically.[21] Variants circulating at<10% are not shown. Note: v1 was not detected in UK based samples, so is excluded from this figure.

A binary indicator variable was used to model the impact in a season where a new genogroup 2 genotype 4 norovirus emerged, classified as 1995/96, 2002/03, 2004/05 and 2005/06. These years were defined based on phylogenetic analysis of norovirus strains,[12], [20] not whether there was an increase in norovirus reports. Since 2002, genogroup 2 genotype 4 noroviruses have been characterised to determine variant diversity based on by partial sequencing of the gene encoding the virus capsid.[21]. Figure 2 (including previously published [13], [21] and recent unpublished data) illustrates the dynamic nature of genogroup 2 genotype 4 noroviruses in England and Wales. The dominance of a new variant in winter is typically presaged by the initial detection of the new virus in the previous spring.

The final regression model thus included terms for relative humidity in the last 35 days, temperature in the last 49 days, population immunity and epidemic seasons controlling for trend, improving diagnostics, background seasonality, bank holidays and weekends. Finally, we allowed for auto-correlation - dependence of each day's count on counts of preceding days, which we expected given the infectious nature of norovirus and because there may be multiple specimens from local outbreaks. Graphical inspection of autocorrelelogram from this model revealed residual correlation between the number of laboratory reports on a given day and the 21 days previous. (Figure S1). Twenty-one autoregressive terms were included to account for this.

The final model was specified as follows:
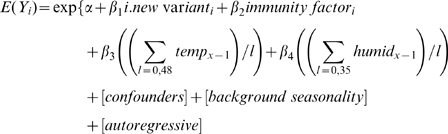



Where 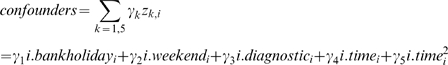


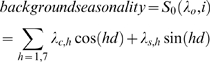


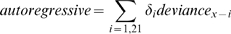
on day *i*, where *Y_i_* is the count of norovirus laboratory reports, *d* is the date in degrees (i*360/365.25) and *h* is the number of Fourier terms.

The AIC was used to determine if a smooth non-linear function of temperature or relative humidity better fit the relationship with norovirus reports, but it was determined that a linear function gave the best fit. The AIC from sequential regression models was also used to determine if there exists a threshold of temperature or relative humidity above or below which there was no effect on norovirus reports. No threshold was found for either weather variable (AIC was minimised with simple linear terms). Therefore, the final model gave daily rates ratios of norovirus reports for new norovirus variants, a 1°C change in temperature, 1% change in relative humidity and 1% change in population immunity. To give a sense for the how the normal range weather fluctuations affects norovirus incidence, the proportion of cases attributable to each weather variable, population immunity and the emergence of epidemic strains was estimated using the methods of Bruzzi.[22] Baseline levels were taken as the 95^th^ percentile value of temperature, and relative humidity and population immunity. Conceptually this means that the calculated attributable fraction (AF) of cases would be averted if temperature, for example, remained at its 95^th^ percentile throughout the year. Therefore, the estimated attributable fraction is sensitive to the choice of baseline.

### Sensitivity analyses

A range of sensitivity analyses were performed to assess the robustness of the results to the construction of the confounder model. A series of different confounder models were fitted: included using year and month terms (model A) or 3, 9 or 12 pairs of Fourier terms (models B, C and D, respectively), in order to determine how sensitive the main results were to how underlying seasonality was accounted for. A version of the final model was configured using weekly instead of daily data, including both outcome and explanatory data (model E). (Figure S2)

We undertook a simulation study to assess the sensitivity of the results to the way ‘new norovirus variant’ seasons were defined. One thousand datasets were simulated whereby exactly four out of 14 years were randomly assigned as ‘new norovirus variant’ seasons. We did this to determine how frequently a significant result (based on the Wald test p-value) would be found by arbitrary selection of ‘new norovirus variant’ seasons in comparison with the empirically defined variable used in the final model. Finally, the association of “reverse lags” of weather variables (e.g. the association of temperature in the *t* days after norovirus reports) was assessed. Since it is not plausible that such associations are causal, their presence would suggest uncontrolled residual confounding.

We then fitted a model to regional level data using as the outcome weekly norovirus counts and using population density weighted mean temperature, mean relative humidity and cumulative rainfall as explanatory weather variables.]Because more laboratory reports come from more populated areas, population density data series were used in order to for weather data to be more representative of populated areas.] In preliminary regional analysis, rainfall again was not significant. The same confounder model was used for each region as was used in the national model. Regional results for temperature (7 weeks lag) and relative humidity (5 weeks lag) were combined in a using meta regression model (with a random intercept for ‘region’).

## Results

**Figure 3:**
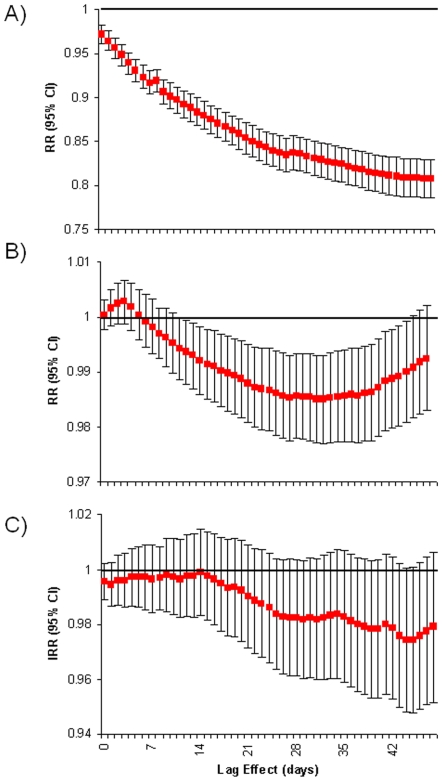
Association of lagged effects of (A) temperature (B) relative humidity and (C) precipitation. Point estimates of rate ratios (red points) and 95% confidence intervals (black lines) are plotted for each day of lag between climate variable and norovirus reports. Presented results are from preliminary models controlling for confounders, secular trends and background seasonality.

**Figure 4:**
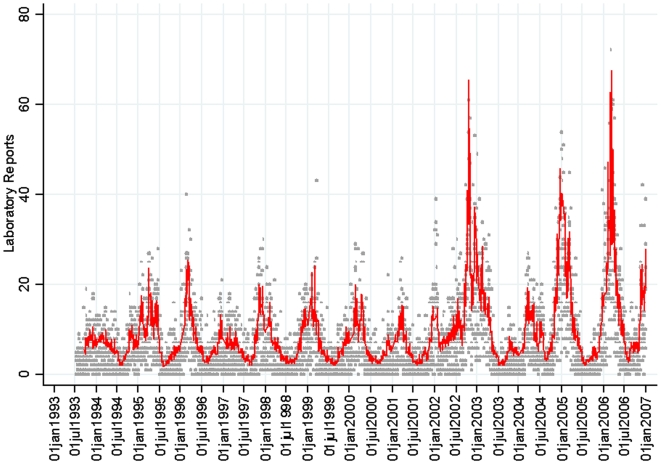
Daily norovirus laboratory reports (grey circles) and predicted values (red line) from full model including temperature, relative humidity, immunity, new variants and autoregressive terms and other confounders.

**Figure 5:**
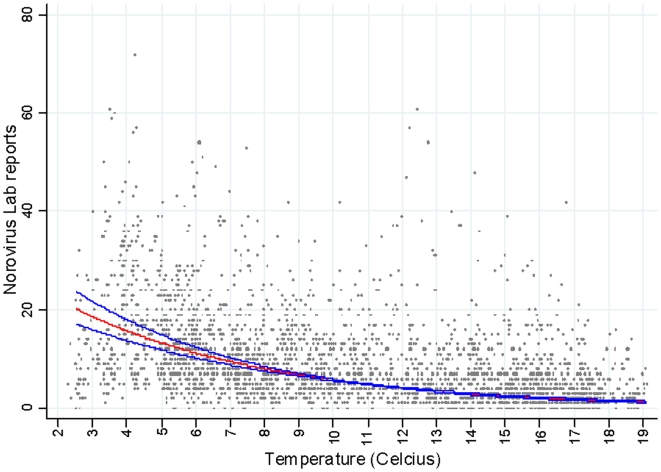
Predicted relationship between temperature and norovirus reports. Predicted relationship (red line) and 95% confidence bounds (blue lines) from full final model including relative humidity, immunity, new variants and autoregressive terms and other confounders.

After controlling for trend, improving diagnostics, background seasonality, bank holidays and weekends, it was found that lower temperature, lower relative humidity, lower population immunity and the emergence of new norovirus variants were independently associated with an increase in norovirus reports (Figure 3, Figure 4). For a 1°C increase in temperature in the previous 49-day period, there was a 15% decrease in norovirus reports (RR = 0.85; 95% CI: 0.83 to 0.86, Figure 5). Temperature in the previous week had the most pronounced effect (RR = 0.95; 95% CI: 0.94 to 0.96) with the effect gradually levelling off to non-significance seven weeks in the past. For a 1% increase in relative humidity in the previous 35-day period, there was a 2% decrease in norovirus reports (RR = 0.98; 95% CI: 0.97 to 0.99). For neither relative humidity nor temperature was a threshold detected above which there was no or reduced effect. Consistent with preliminary analysis, cumulative recent rainfall was not associated with norovirus incidence (RR = 0.98; 95% CI: 0.96 to 1.01 for previous 28 days, similar results (not presented) from 0 to 49 days). Comparatively, temperature had a greater effect as change from the 90^th^ to the 10^th^ temperature centile (16.8 to 5.0°C) corresponds to a seven-times increase in the rate of norovirus reports (RR = 7.2; 95% CI: 5.8 to 9.1). A change in relative humidity from the 90^th^ to the 10^th^ centile (87% to 68%) corresponded to a much smaller effect on norovirus (RR = 1.4; 95% CI: 1.3 to 1.6).

The emergence of a new variant (in the 4 years described above) was associated with a 16% daily increase in norovirus reports (RR = 1.16; 95% CI: 1.10 to 1.22). The level of population immunity was inversely associated with numbers of norovirus reports; the previous season being 25% larger than normal (population immunity factor = 1.25) was associated with a 6.6% decrease in cases (RR = 0.94; 95% CI: 0.93 to 0.96).

Temperature variation was associated with the largest attributable fraction: 60% of cases. Relative humidity, immunity levels and emergence of new strains were the cause of comparatively fewer overall cases (AF = 18%, 13% and 5%, respectively).

**Figure 6:**
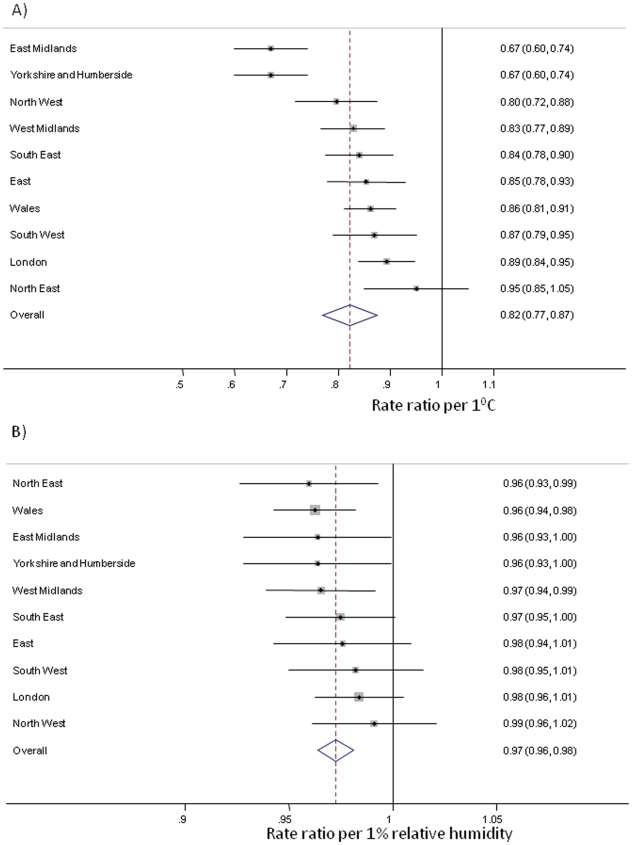
Forest plot of regional and pooled estimates of the relationship between (A) temperature and (B) relative humidity with norovirus reports. Regional RR estimates (box and horizontal line) and national pooled estimate (diamond) from random effects model are for 1°C and 1% relative humidity controlling for all other weather, confounding variables in the final regression model.

Weather results were highly consistent in the individual regions of England and Wales. In all regions there was an inverse relationship, with 9 out of 10 regions significant at p<0.05; the combined effect estimate was (RR = 0.82; 95% CI: 0.77 to 0.87, Figure 6a). In all regions there was also an inverse relationship with mean relative humidity (RR = 0.972; 95% CI: 0.964 to 0.981, Figure 6b).

None of the weather, immunity or viral results were sensitive the way background seasonality was modelled (Figure S2). Model coefficients for variables of interest were very similar if the model was applied to weekly instead of daily data. Reverse lags (temperature in the days after rather than before norovirus reports) had no association. If such an association was found, we would have suspected that the weather variables were just indicators of the seasonality of disease. The finding of a clear association with disease in the near future is evidence suggesting a genuine effect. New variant seasons were defined randomly and 1000 simulated dataset were created. The ‘new variant’ variable was significant at the p<0.05 level in 21% of the simulations, at the p<0.01 level in 7% of the simulations and at the p<0.001 level in none of the simulations. No simulations reached the level of significance when the variable was defined empirically as years when new variants were detected (p<0.0001).

## Discussion

Increases in norovirus are associated with cold, dry temperature, low population immunity and the emergence of novel genogroup 2 genotype 4 antigenic variants. This is the first study to use rigorous statistical methods to demonstrate an independent association of norovirus infections with weather factors, host population and viral evolution. Using this approach, it has been possible to quantify the epidemiological impact of the emergence of a new variant and a change in population immunity.

These methods provide an estimate of the independent effect of each factor of interest. However, population immunity and viral evolution are clearly are not independent phenomena. Genogroup 2 genotype 4 viruses evolve under positive selective pressure,[12] so when population immunity is high, there is an increased likelihood that a new variant will emerge. Despite this, these two factors had significant effects after controlling for the other, but there was not a statistical interaction between them.

Although these time-series adapted regression models estimate the short term effects of weather factors, they may also provide insight into the underlying regular seasonal pattern. If cold dry weather explains the deviation from the ‘normal’ pattern, then it is likely that these factors also contribute to the underlying regular seasonality. We found no evidence of a threshold above or below which the weather variables had no effect. This suggests norovirus is affected incrementally across the range of humidity and temperature that occurs in England and Wales. These methods account for lags in seasonality and other confounders. In terms of defining new variant years, we have taken a long term approach, defining these seasons based on molecular surveillance data. Previous studies have looked at specific years when new strains have emerged and there have been an associated surge in cases. The large 2002/03 epidemic was probably associated with an immune escape variant.[12], [13], [23] This analysis suggests that the emergence of new variants is associated with an increase in cases, though not necessarily always as dramatic as the 2002/03 season.

Certain limitations of the data and model deserve consideration. Firstly, only a small selected set of weather factors were considered. Other factors, such as UV in daylight, may well be important in affecting transmission, but are highly localised and therefore may not correlate well with national data. Indeed, relative humidity, which is inversely associated with norovirus reports, may vary substantially across regions. However, analysis at the regional level produced highly consistent results for temperature and relative humidity. Rainfall, which is also highly localised, was not associated with norovirus incidence either in preliminary analysis or in the full model. This suggests that either there is no relationship between normal fluctuations in rainfall and norovirus transmission or this level of analysis did not capture local patterns or extreme rainfall events that may contribute to transmission through flooding, for example. Secondly, there are important limitations with the time-series of norovirus reports. The weekly counts fluctuate considerably, particularly in early years when overall numbers were smaller. Whether the increase of reports since 2002 represents a real emergence or is a result of improved sampling and diagnostics is unknown. In either case, our results are not affected by long term temporal trends since they have been controlled for in the confounder model. Thirdly, the molecular data used to define new variants has improved substantially in recent years, as more groups around the world have begun typing and sharing data.(e.g. [24]) Despite this, there is debate about what molecular or antigenic changes confer an important new variant.[25] It is possible that the long gap between 1996 and 2001 when no new variants appear to emerge is due to limited typing data being available from that period. Again, the results are robust to this uncertainly; one thousand simulations where ‘new variant seasons’ were randomly chosen never produced a result to the level of statistical significance when defined based on molecular data. Fourth, we have assumed that population immunity is function of the cumulative number of cases in the previous year. Therefore, immunity levels vary between years but not within them. In reality, immunity is at a low point sometime at the beginning of the epidemic season and peaks sometime towards the end. Modelling this directly, either in a statistical model or in a transmission model, requires knowledge of immunity in the population. The duration of immunity is probably less than 1 year,[26], [27] but the exact period of waning is not known. Furthermore, immunity will not just be a function of cases but will also be influenced by asymptomatic infection, which the incidence and level of immunity conferred is also unknown. There is limited cross-protective immunity to noroviruses genotypes within the same genogroup but little between genogroups,[28]–[30] so actual population immunity will depend both on the antigens recently encountered and the ones currently circulating. Immunity to genogroup 2 genotype 4 viruses evolve novel antigens which may escape immunity.[12] We model these two processes separately: we include a parameter representing overall levels of population immunity overall and a parameter indicating years when novel genogroup 2 genotype 4 viruses emerged. The approach taken here allows these unknown quantities to be absorbed in the background seasonal pattern (by the Fourier terms); just the impact of year on year fluctuations was investigated. The finding that both the immunity factor and the new variant indicator are highly significant suggests the importance of both mechanisms. Finally, it is important to note that – although the final model was fairly complicated, only four parameters were of interest (out of an initial 5, including rainfall). The rest of the model parameters were included to control for confounding, secular trends, background seasonality or autocorrelation in the data. The four parameters of interest were highly significant (p<0.0001) suggesting multiple testing in model construction was not a major issue.

Originally called “winter vomiting disease”,[31] norovirus gastroenteritis has long been associated with a cold weather seasonality in temperate climates.[32] However, previous studies on norovirus have not attempted to identify the effect of specific climate variables independent of other seasonal trends. A number of enteric and respiratory viruses, most notably rotavirus and influenza A, also exhibit strong winter-time seasonality in temperate climates. Although the factors underlying transmission of these viruses have not been fully characterised, they have been studied more extensively than noroviruses. In the tropics, a systematic review has identified that rotavirus incidence is highest in periods of cool and dry weather,[33] although these findings are not universal [34], [35] Ambient temperature is thought to be driver of influenza seasonality,[36] although it is difficult to determine whether temperature itself affects transmission,[37] or whether it is a driver of seasonal behaviours like crowding, indoor heating and air travel.[38] The most common enteric bacterial pathogens in developed countries (*Salmonella* and *Campylobacter*) are largely zoonotic, rather than directly transmitted and tend to be associated with higher temperature and wet climatic conditions.[39]–[42]

For viruses that are transmitted directly from person to person or through local droplet/fomite contamination, survival in the environment may play a key role in transmissibility. Noroviruses cannot be cultured *in vitro*, so studies cannot directly examine virus survival under different conditions. Surrogate pathogens, including feline and murine calciviruses are inactivated by relatively extreme UV heat and high pressure.[43], [44]. Feline calicivirus survival is shorter in 25°C and warmer in water compared with at 4°C.[45] There is no animal model of norovirus. In a unique study on aerosol spread in guinea pigs, influenza has been shown to be more transmissible under cold and dry conditions.[10] Aerolsolization of virus particles in droplets or fomites following a vomiting event is an important feature of norovirus transmission [46], [47] and therefore may be sensitive to similar environmental drivers as influenza. Polio, an enterovirus, survives better in conditions of high relative humidity.[48]

The results of the present study point to the potential value of incorporating multiple information sources into a norovirus early warning system. In temperate, developed countries the severe health and economic impact of norovirus occurs in healthcare facilities.[3] Early detection of emerging variants may allow healthcare facilities to prepare for increased winter time burden, especially when other conditions are conducive to norovirus spread.

Due to short lived immunity, high viral diversity and multiple routes of transmission, norovirus epidemiology is complex. Here, cool and dry weather, population immunity and viral evolution are identified as the drivers of these complicated patterns. Further studies, employing similar methodology, should determine whether the same factors underlie norovirus epidemiology in other temperate and tropical settings.

## Supporting Information

Figure S1 (0.06 MB PDF)

Figure S2 (0.06 MB PDF)
