# Supplementary material for: Host, Weather and Virological Factors Drive Norovirus Epidemiology: Time-Series Analysis of Laboratory Surveillance Data in England and Wales
Source: PLoS One. 2009 Aug 24;4(8):e6671. doi: 10.1371/journal.pone.0006671 (PMC2726937; doi:10.1371/journal.pone.0006671)

Figure S1. Partial autocorrelation of deviance residuals up to forty days. Twenty-one autoregressive terms were included in the final model.

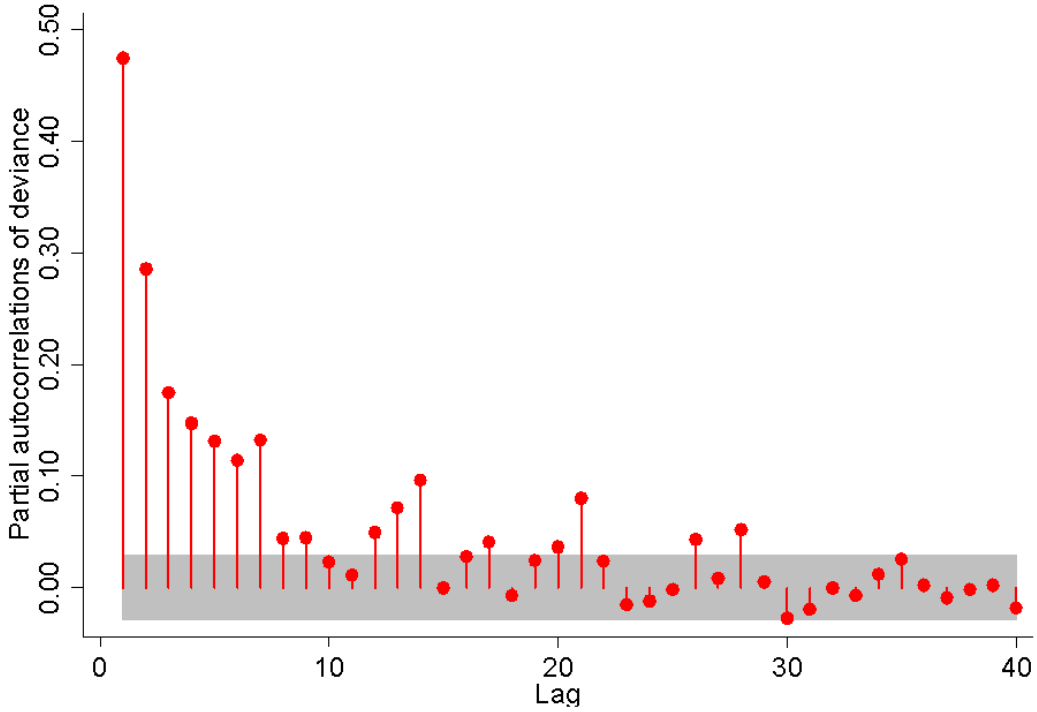

Supplement: Figure S1 — (0.06 MB PDF) [file pone.0006671.s001.pdf]
