# Supplementary material for: Host, Weather and Virological Factors Drive Norovirus Epidemiology: Time-Series Analysis of Laboratory Surveillance Data in England and Wales
Source: PLoS One. 2009 Aug 24;4(8):e6671. doi: 10.1371/journal.pone.0006671 (PMC2726937; doi:10.1371/journal.pone.0006671)

Figure S2. Sensitivity analysis. Rate ratios of temperature, relative humidity, new variants and immunity in the Final Model, model including year and month indicator variables (Model A), three , nine and twelve Fourier terms (Models B, C and D) and modelling weekly instead of daily data (Model E).

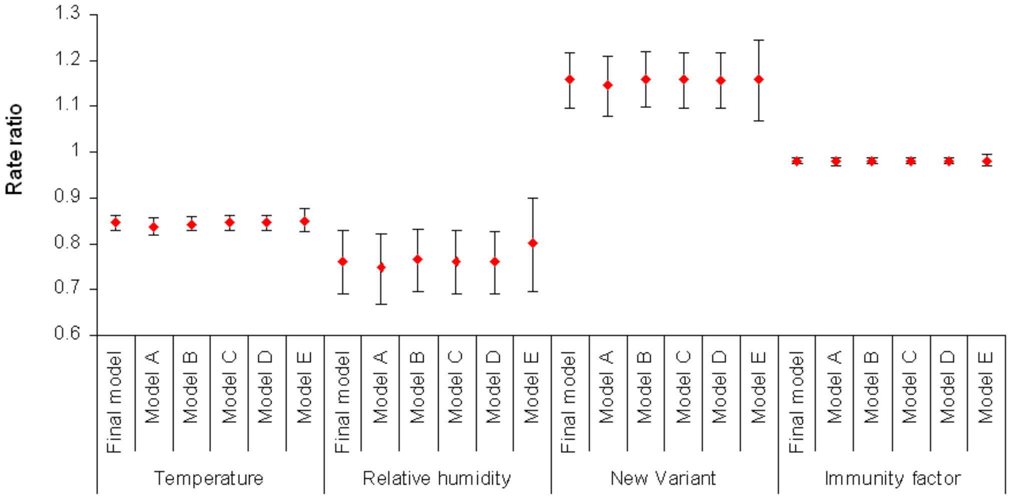

Supplement: Figure S2 — (0.06 MB PDF) [file pone.0006671.s002.pdf]
